# Associations of TV Viewing Duration, Meals and Snacks Eaten When Watching TV, and a TV in the Bedroom with Child Adiposity

**DOI:** 10.1002/oby.22288

**Published:** 2018-09-30

**Authors:** Paul J. Collings, Brian Kelly, Jane West, John Wright

**Affiliations:** ^1^ Bradford Institute for Health Research, Bradford Teaching Hospitals NHS Foundation Trust Bradford UK; ^2^ Department of Health Sciences University of York UK

## Abstract

**Objective:**

This study aimed to examine the associations of TV parameters with adiposity in early life.

**Methods:**

Data were collected as part of the Born in Bradford (BiB) longitudinal birth cohort study. Child TV viewing duration was parent reported, and BMI, the sum of triceps and subscapular skinfolds, and waist circumference were measured at ~12, 18, 24, and 36 months of age in 1,338 children. Mixed effects models were used to quantify adjusted associations of TV viewing duration with adiposity markers, incorporating data from all time points. Linear regression was used to investigate differences in adiposity levels across frequencies of eating meals and snacks while watching TV at age ~24 months and between children who did and did not have a TV in their bedroom at age ~36 months.

**Results:**

Every 1 h/d of TV viewing was associated with a 0.075‐cm larger (95% CI: 0.0034‐0.15) waist circumference, independent of covariates including sleep duration, dietary factors, and physical activity level. There was no evidence for any other associations.

**Conclusions:**

TV viewing duration is independently associated with abdominal adiposity in young children. Limiting TV viewing from an early age may be important for primary prevention of obesity.

## Introduction

Sedentary time comprises all low‐energy‐expenditure sitting, reclining, and lying behaviors that are performed while awake 1. Objective estimates show that young children worldwide are highly sedentary 2, with children living in the United Kingdom spending between one‐third and up to one‐half of their waking day (on average 14 hours) in sedentary behavior 3, 4, 5. Questions remain as to whether total sedentary time has a meaningful impact on early childhood health and well‐being, but the growing consensus is that specific sedentary behaviors, particularly TV viewing, may be detrimental 6, 7, 8.

Despite increased home ownership of multimedia devices, TV viewing on a TV set remains the dominant screen time behavior in early childhood 9, 10. A recent study reported that UK children aged around 6 months watched nearly 1 hour of TV per day, and viewing time more than doubled to exceed 2 hours daily by 36 months of age 11. Similar levels of TV viewing have been documented in young children internationally 12. An association (albeit small in magnitude) has consistently been reported between TV viewing duration and higher child adiposity 6, 7, 8. This would appear plausible as, by definition, sedentary behavior involves low energy expenditure. However, given that studies of total sedentary time have tended to yield null associations 6, 7, 8, it is conceivable that TV viewing may be associated with higher adiposity not because of accumulated sedentary time per se, but because of coexisting (possibly mediating) obesogenic diet, inactivity, and sleep behaviors 13. To date, few studies have examined independent associations or mediation by these factors when exploring TV viewing and childhood adiposity, and the few investigations that have been performed provide mixed results 7, 13. Additional studies are warranted to quantify associations between TV viewing duration and child adiposity while considering the roles of diet, physical activity, and sleep parameters 7. This will improve knowledge regarding the independence of associations and possible underlying pathways of action, and it could help to identify early opportunities for obesity prevention.

The context of TV viewing is also important. The presence of a TV set in a child’s bedroom has been related to higher childhood BMI and overweight risk independently of TV viewing time 14, 15, as has eating while watching TV in studies of school‐aged children 16. It remains the case, however, that relatively few studies have investigated parameters beyond TV viewing duration, and research is particularly lacking in children from minority ethnic groups and deprived backgrounds. These children are at high risk for overweight and obesity 17, 18, and they watch more TV, eat more frequently while watching TV, and more commonly have a TV in their bedroom 11, 19.

The aim of this study was to investigate independent associations of multiple TV parameters with adiposity in early life. The following exposures were examined: (1) TV viewing duration, (2) meals and snacks eaten while watching TV, and (3) the presence of a TV set in a child’s bedroom.

## Methods

### Participants

The Born in Bradford (BiB)‐1000 study is nested within the BiB birth cohort with the purpose of investigating modifiable risk factors for childhood obesity 20. Full recruitment details and measurement procedures have previously been described 21, 22. Pregnant women (*n* = 1,916) at 26 to 28 weeks gestation were invited to the BiB‐1000 study while attending routine hospital appointments in 2008 and 2009; 1,735 (90.6%) women accepted the invitation. Consent to medical records access was given, and periodic postnatal follow‐ups (when offspring were ~6, 12, 18, 24, and 36 months old) were conducted by bilingual trained researchers. Compliance at each measurement time point ranged between 70% to 77%, and 47% of participants attended every time point. The BiB‐1000 cohort is comparable to the larger BiB cohort, which is representative of Bradford, the sixth largest and one of the most deprived and ethnically diverse cities in the United Kingdom 20, 21. Data were collected between 2008 and 2013. Ethical approval for all aspects of the study was granted by Bradford Research Ethics Committee (Reference 07/H1302/112).

This investigation included only children from singleton births with data for TV viewing, adiposity, and potential confounders from the 12‐month visit and onward (*n* = 1,411), as we have previously shown that TV viewing patterns take approximately 12 months to consolidate 11. Children with a mother belonging to an ethnic group other than South Asian (Pakistani, Indian, and Bangladeshi) or white (British or other, e.g., white European) were also excluded (remaining *n* = 1,338). Missing data comparisons have found few differences in the children included in this study compared with those excluded 22.

### TV parameters

Children’s average daily DVD and TV viewing durations were parent reported at all time points (when children were aged ~12, 18, 24, and 36 months) in answer to a disaggregated question pertaining to the type of day (week or weekend) and whether the behavior was before or after 6 pm 11. Weighted mean daily TV viewing before and after 6 pm was calculated ([{weekday hours × 5} + {weekend hours × 2}] / 7) and combined to estimate total daily TV viewing (hours per day). For description, mean TV viewing duration across all measurement occasions was calculated and split into approximate quartiles (<0.75; 0.75 to < 1.25; 1.25 to < 2; and ≥ 2 h/d).

At the 24‐month time point only, parents reported how often in the last month their child had watched TV at meal times and had eaten snacks while watching TV. The data were collapsed to three groups: never or rarely (≤1‐3 times in the last month), sometimes (1‐4 times per week), and often (≥ 5 times per week). Information was further collated (yes/no) about whether children usually ate breakfast, lunch, or dinner in front of the TV. Parents reported whether their child had a TV set in the bedroom (yes/no) at the 36‐month visit. Supporting Information Figures S1, S2, and S3 show the exact questions put to parents alongside possible responses.

### Adiposity measurements

At all visits, children’s weight and height were assessed by trained researchers, and BMI (kilograms per meter squared) was calculated. Waist circumferences were measured at the level of the navel. Skinfolds of the left triceps and subscapular were measured to the nearest 0.1 mm and were combined to their total sum. Reliability metrics indicated good intra‐ and interobserver technical error for measurements 5.

### Covariates

Three socioeconomic status (SES) groups were synthesized from information collated during interviews with parents (usually mothers) about their education, employment, housing tenure, financial situation, and ownership of goods at the point of recruitment 23. As part of the interviewer‐administered recruitment questionnaire, maternal ethnicity (used as a proxy for child ethnicity), age, and smoking during pregnancy were also reported, and maternal height and weight were measured; weight was remeasured at the 24‐ and 36‐month time points and maternal BMI calculated. Child gender was extracted from neonatal medical records. At the 24‐month time point, parents reported their child’s bedtime regularity and moderate‐to‐vigorous physical activity level 24, for which information was further available at the 36‐month visit. Child sleep duration, consumption of unhealthy snacks and drinks (frequency of biscuit, crisps, cake, sweets, chocolate, sugar‐sweetened beverage consumption), and daily fruit and vegetable portions were parent reported at all visits 22. Other plausible covariates (including number of previous births, child gestational age, birth weight, breastfeeding history, and age introduced to solid foods) were not used in analyses as they did not consistently vary by TV parameters and entailed missing data.

### Statistics

#### Associations of TV viewing duration with adiposity using data from all time points.

Participant characteristics were first summarized across quartiles of average TV viewing duration and compared using trend and χ^2^ tests. For the main analysis, each adiposity variable (modeled continuously) was first individually regressed against age. Models including age, age squared, and age cubed were most suited to describe the time trend in adiposity. To efficiently analyze the repeated‐measures data, which entailed some missingness because of nonattendance at particular visits and difficulties in acquiring waist and skinfold measurements, mixed effects regression models were used. This technique permitted inclusion of all available exposure and outcome data (up to four of each per child). Following preliminary models that included only age (and age squared and cubed), gender, ethnicity, and child height (not applicable to models with BMI as the outcome), Model 1 was further adjusted for SES, maternal pregnancy age, maternal smoking in pregnancy, and maternal early pregnancy BMI. Thereafter, to elucidate heterogeneity of associations by age, an interaction term was added, and the main effects of TV viewing on adiposity markers at age 18, 24, and 30 months (at which the bulk of data were concentrated) were calculated (Model 1a). In Model 2, sleep duration, unhealthy snacking, and fruit and vegetable intake were added as potential confounding or mediating factors (data for these variables were collected at all time points and were incorporated in the analyses as time‐varying factors). The results represent differences in adiposity markers for every 1 h/d of TV viewing. There was no consistent evidence for effect modification by ethnicity, so the results are presented for the whole group combined; there was no evidence for curvilinear associations when quadratic terms for TV viewing duration were added to models (data not shown).

#### Associations of eating meals and snacks when watching TV with adiposity at the 24‐month time point.

Linear regression models were used to investigate differences (presented as estimated marginal mean [95% CI]) in adiposity markers at ~24 months of age between the three groups (never or rarely [reference category], sometimes, and often) for eating meals and snacks when watching TV. Model 1 was adjusted for age, gender, ethnicity, height (not applicable to models with BMI as the outcome), SES, maternal age, maternal smoking during pregnancy, and concurrent (in this case, the 24‐month time point) maternal BMI. Model 2 was further adjusted for concurrent TV viewing duration, sleep duration, and bedtime regularity. Habitual unhealthy snacking and daily fruit and vegetable intake were added as covariates in a final model.

#### Associations of a TV set in the child’s bedroom with adiposity at the 36‐month time point.

Similarly, linear regression models were used to test for differences (presented as estimated marginal mean [95% CI]) in adiposity markers at ~36 months of age between children with and without a TV set in their bedroom. Model 1 was constructed identically to the analyses for eating when watching TV. Model 2 was further adjusted for concurrent (reported at the 36‐month time point) TV viewing duration, sleep duration, habitual unhealthy snacking, and fruit and vegetable intake.

All statistical analyses were conducted in Stata Statistical Software version 13.1 (StataCorp, College Station, Texas), and two‐tailed *P* < 0.05 was deemed statistically significant. The following sensitivity analyses were performed: (1) total sleep duration was replaced as a covariate with the hours slept only after 6 pm; (2) models were additionally adjusted for physical activity, which entailed substantial missing data; and (3) all models with BMI as the outcome were replaced with BMI *z* scores as the dependent variable 25.

## Results

### Participant characteristics

The entire sample included 1,338 children (58.0% South Asian origin; 48.7% boys) who provided up to 3,829 measurement observations; complete data at all time points were available for 399 children. Overall, the average TV viewing duration was 1.3 (1.2) h/d, of which 0.3 (0.5) h/d were viewed after 6 pm. Participant characteristics by TV viewing quartiles are shown in Table 1. South Asian children and girls watched more TV than white children and boys, respectively. In general, children who watched more TV were from more deprived families, consumed more unhealthy snacks and fewer fruits and vegetables, and slept more before 6 pm but fewer hours after 6 pm.

**Table 1:** Participant characteristics stratified by quartiles of average TV viewing duration using data from all time points

			**TV viewing (h/d) quartiles**			
	***n***	**Overall**	**<0.75 (*n *= 315)**	**0.75 to <1.25 (*n *= 375)**	**1.25 to <2 (*n *= 348)**	**≥2 (*n *= 300)**
**Gender, *n* (%) boys**	1,338	652 (48.7)	**171 (54.3)**	**194 (51.7)**	**144 (41.4)**	**143 (47.7)^a^**
**Ethnicity, *n* (%) South Asian**	1,338	776 (58.0)	**152 (48.3)**	**209 (55.7)**	**203 (58.3)**	**212 (70.7)^b^**
**Socioeconomic status, *n *(%)**	1,338					
**Least deprived**		506 (37.8)	**137 (43.5)**	**155 (41.3)**	**130 (37.4)**	**84 (28.0)**
**Moderately deprived**		620 (46.3)	**124 (39.4)**	**172 (45.9)**	**166 (47.7)**	**158 (52.7)**
**Most deprived**		212 (15.8)	**54 (17.1)**	**48 (12.8)**	**52 (14.9)**	**58 (19.3)^b^**
**Maternal pregnancy age (y)**	1,338	27.1 ± 5.6	**27.1 ± 5.5**	**28.0 ± 5.8**	**27.2 ± 5.7**	**26.0 ± 5.2^a,c^**
**Smoked in pregnancy, *n* (%)**	1,338	212 (15.8)	53 (16.8)	56 (14.9)	57 (16.4)	46 (15.3)
**Maternal early pregnancy BMI (kg/m^2^)**	1,338	24.8 (7.3)	24.4 (7.7)	25.1 (7.5)	25.5 (7.2)	24.0 (6.9)
**Unhealthy snacks (*n*, weekly)**	1,338	11.8 (10)	**9 (8.5)**	**10.9 (9.3)**	**12.6 (9.0)**	**13.9 (11.5)^b,c^**
**Fruits and vegetables (daily portions)**	1,338	4.7 ± 1.9	**5.0 ± 2.0**	**4.7 ± 1.8**	**4.6 ± 1.7**	**4.6 ± 1.9^a,c^**
**TV viewing (h/d)**	1,338	1.3 (1.2)	**0.5 (0.4)**	**1.0 (0.3)**	**1.6 (0.3)**	**2.7 (1.0)^b,c^**
**Before 6 pm**	1,338	0.9 (0.9)	**0.3 (0.3)**	**0.7 (0.4)**	**1.2 (0.4)**	**1.9 (0.8)^b,c^**
**After 6 pm**	1,338	0.3 (0.5)	**0 (0.2)**	**0.3 (0.4)**	**0.5 (0.5)**	**0.9 (0.8)^b,c^**
**Sleep duration (h/d)**	1,338	12.5 ± 1.1	12.6 ± 1.2	12.5 ± 1.1	12.5 ± 1.1	12.4 ± 1.2
**Before 6 pm**	1,338	3 (1.9)	**2.6 (1.8)**	**2.9 (2)**	**3 (2)**	**3.5 (1.7)^b,c^**
**After 6 pm**	1,338	9.4 ±** **1.2	**9.7 ± 1.3**	**9.5 ± 1.2**	**9.4 ± 1.2**	**8.9 ± 1.2^b,c^**
**Physical activity (h/d)**	1,107	2.8 (2.1)	**3.1 (2.0)**	**2.7 (2.3)**	**2.8 (2.1)**	**2.5 (1.8)^d^**

For categorical variables, *P* values are from χ^2^ tests. For continuous variables, values are mean ± SD or median (interquartile range) given skewness, and *P* values are from trend tests across TV viewing quartiles (regression, skewed variables natural log transformed). Values for maternal BMI, diet, sleep, TV viewing, and physical activity variables represent averages derived from all data collected when mothers were recruited and when children were about 12, 18, 24, or 36 months old. Significant differences are highlighted in bold. South Asian ethnicity includes Pakistani (*n *= 688), Indian (*n *= 60), and Bangladeshi (*n *= 28); white ethnicity includes British (*n *= 535) and other white (*n *= 27). Socioeconomic status groups correspond with Fairley et al. (23) as follows: least deprived (least socioeconomically deprived, most educated and employed, not materially deprived), moderately deprived (employed, no access to money and benefits, not materially deprived), and most deprived (most economically deprived).

^a^ *P *< 0.01.

^b^ *P *< 0.001.

^c^Group differences persisted following adjustment for gender, ethnicity, and socioeconomic status.

^d^ *P *< 0.05.

Table 2 provides details of 798 children aged ~24 months for whom we had data for eating habits when TV viewing. The proportion of children who never or rarely ate meals while watching TV was 44.4%, whereas 24.9% of children sometimes and 30.7% of children often consumed meals while watching TV. Approximately one‐third of children never or rarely (31.9%), sometimes (37.0%), and often (31.1%) snacked while TV viewing, respectively. A higher proportion of South Asian children than white children ate when watching TV. Children who ate more frequently when TV viewing consumed more unhealthy snacks, slept more before 6 pm but fewer hours after 6 pm, and watched more TV throughout the day.

**Table 2:** Participant characteristics stratified by eating meals and snacks when watching TV at the 24‐month time point

			**Meals eaten watching TV**			**Snacks eaten watching TV**		
	***n***	**Overall**	**Never/rarely (*n *= 354)**	**Sometimes (*n *= 199)**	**Often (*n *= 245)**	**Never/rarely (*n *= 255)**	**Sometimes (*n *= 295)**	**Often (*n *= 248)**
**Age (month)**	798	25.0 (1.2)	25.1 (1.2)	24.9 (1.2)	25.0 (1.1)	25.1 (1.2)	24.9 (1.1)	25.0 (1.3)
**Gender, *n* (%) boys**	798	395 (49.5)	171 (48.3)	106 (53.3)	118 (48.2)	123 (48.2)	160 (54.2)	112 (45.2)
**Ethnicity, *n* (%) South Asian**	798	439 (55.0)	**162 (45.8)**	**106 (53.3)**	**171 (69.8)^a^**	**124 (48.6)**	**149 (50.5)**	**166 (66.9)^a^**
**Socioeconomic status, *n *(%)**	798							
**Least deprived**		319 (40.0)	**145 (41.0)**	**98 (49.3)**	**76 (31.0)**	100 (39.2)	134 (45.4)	85 (34.3)
**Moderately deprived**		350 (43.9)	**150 (42.4)**	**76 (38.2)**	**124 (50.6)**	109 (42.8)	117 (39.7)	124 (50.0)
**Most deprived**		129 (16.2)	**59 (16.7)**	**25 (12.6)**	**45 (18.4)^b^**	46 (18.0)	44 (14.9)	39 (15.7)
**Maternal pregnancy age (y)**	798	27.3 ± 5.5	27.5 ± 5.4	27.2 ± 5.8	27.0 ± 5.3	27.4 ± 5.7	27.2 ± 5.3	27.3 ± 5.4
**Smoked in pregnancy, *n* (%)**	798	125 (15.7)	**66 (18.6)**	**34 (17.1)**	**25 (10.2)^c^**	**44 (17.3)**	**55 (18.6)**	**26 (10.5)^c^**
**Maternal BMI (kg/m^2^)**	798	26.1 (8.0)	26.1 (7.6)	26.4 (8.3)	25.8 (7.7)	26.4 (7.8)	26.2 (8.6)	25.5 (7.3)
**Unhealthy snacks, *n*, weekly**	798	11 (12)	**10 (11)**	**12 (11.5)**	**13 (14)^b,d^**	**10 (10.5)**	**10.5 (11.5)**	**15 (16.3)^a,d^**
**Fruits and vegetables (daily portions)**	798	4.3 ± 2.2	**4.5 ± 2.2**	**4.5 ± 2.3**	**3.9 ± 2.0^a^**	**4.4 ± 2.2**	**4.5 ± 2.3**	**4.0 ± 2.0^c^**
**TV viewing (h/d)**	798	1.3 (2)	**1 (1.1)**	**1.5 (1.6)**	**2 (2)^a,d^**	**0.7 (1)**	**1.5 (1.7)**	**2 (2)^a,d^**
**Before 6 pm**	798	0.8 (1)	**1 (1.1)**	**1.5 (1.6)**	**2 (2)^a,d^**	**0.5 (1.4)**	**1.1 (1)**	**1.5 (1.7)^a,d^**
**After 6 pm**	798	0.5 (0.5)	**0 (0.5)**	**0.5 (0.5)**	**0.5 (1.5)^a,d^**	**0 (0.5)**	**0.5 (0.5)**	**0.5 (1.2)^a,d^**
**Regular bedtime, *n* (%)**	798	553 (69.3)	**257 (72.6)**	**121 (60.8)**	**175 (71.4)^c,d^**	181 (71.0)	192 (65.1)	180 (72.6)
**Sleep duration (h/d)**	791	12.4 ± 1.4	12.4 ± 1.2	12.3 ± 1.4	12.5 ± 1.6	12.4 ± 1.4	12.4 ± 1.3	12.4 ± 1.5
**Before 6 pm**	791	3 (2)	**2.5 (1.5)**	**2.5 (2)**	**3 (2.5)^a,d^**	**2.5 (2)**	**2.5 (2.3)**	**3 (2)^a,d^**
**After 6 pm**	791	9.4 ± 1.6	**9.7 ± 1.5**	**9.4 ± 1.6**	**9.0 ± 1.6^a,d^**	**9.6 ± 1.5**	**9.6 ± 1.5**	**9.0 ± 1.6^a,d^**
**Physical activity (h/d)**	624	2.6 (1.5)	2.7 (1.5)	2.4 (1.5)	2.7 (1.5)	2.7 (1.6)	2.6 (1.5)	2.6 (1.4)

For categorical variables, *P *values are from χ^2^ tests. For continuous variables, values are mean ± SD or median (interquartile range) given skewness, and *P* values are from ANOVA or Kruskal‐Wallis tests as appropriate. Values for maternal BMI, diet, sleep, TV viewing, and physical activity are from data collected when children were about 24 months old. Significant differences are highlighted in bold. South Asian ethnicity includes Pakistani (*n *= 390), Indian (*n *= 33), and Bangladeshi (*n *= 16); white ethnicity includes British (*n *= 339) and other white (*n *= 20). Socioeconomic status groups correspond with Fairley et al. (23) as follows: least deprived (least socioeconomically deprived, most educated and employed, not materially deprived), moderately deprived (employed, no access to money and benefits, not materially deprived), and most deprived (most economically deprived).

^a^ *P *< 0.001.

^b^ *P *< 0.01.

^c^ *P *< 0.05.

^d^Group differences persisted following adjustment for ethnicity and socioeconomic status.

Table 3 summarizes the characteristics of 909 children aged ~36 months for whom information about a TV set in the bedroom was available. Nearly one‐quarter (24.3%) of all children had a TV in their bedroom, but the prevalence was lower in South Asian children (14.9%) than white children (37%). Children with a TV set in their bedroom watched more TV after 6 pm and ate fewer fruits and vegetables.

**Table 3:** Participant characteristics stratified by presence of a TV set in child’s bedroom at the 36‐month time point

			**TV set in bedroom**	
	***n***	**Overall**	**No (*n *= 688)**	**Yes (*n *= 221)**
**Age (month)**	909	36.8 (1.1)	36.8 (1.1)	36.9 (1)
**Gender, *n* (%) boys**	909	427 (47.0)	323 (47.0)	104 (47.1)
**Ethnicity, *n* (%) South Asian**	909	522 (57.4)	**444 (64.5)**	**78 (35.3)^a^**
**Socioeconomic status, *n *(%)**	909			
**Least deprived**		366 (40.3)	**296 (43.0)**	**70 (31.7)**
**Moderately deprived**		401 (44.1)	**296 (43.0)**	**105 (47.5)**
**Most deprived**		142 (15.6)	**96 (14.0)**	**46 (20.8)^b^**
**Maternal pregnancy age (y)**	909	27.4 ± 5.6	**27.7 ± 5.4**	**26.3 ± 5.9^a,c^**
**Smoked during pregnancy, *n* (%)**	909	129 (14.2)	**67 (9.7)**	**62 (28.0)^a,c^**
**Maternal BMI (kg/m^2^)**	909	26.4 (7.6)	**26.2 (7.1)**	**27.2 (9.1)^d^**
**Unhealthy snacks, *n*, weekly**	909	15 (17)	15 (16.5)	16 (16.5)
**Fruits and vegetables (daily portions)**	909	5.0 ± 2.8	**5.1 ± 2.8**	**4.5 ± 2.8^b,c^**
**TV viewing (h/d)**	909	2.1 ± 1.5	**2.0 ± 1.5**	**2.3 ± 1.5^c,d^**
**Before 6 pm**	909	1.5 ± 1.1	1.5 ± 1.1	1.6 ± 1.1
**After 6 pm**	909	0.6 ± 0.7	**0.5 ± 0.7**	**0.7 ± 0.8^b,c^**
**Sleep duration (h/d)**	909	11.8 ± 1.2	11.9 ± 1.2	11.8 ± 1.1
**Before 6 pm**	909	2.3 ± 1.6	**2.4 ± 1.6**	**2.1 ± 1.6^d^**
**After 6 pm**	909	9.5 ± 1.4	9.5 ± 1.4	9.7 ± 1.5
**Physical activity (h/d)**	785	2.7 (1.5)	2.7 (1.5)	2.7 (1.5)

For categorical variables, *P* values are from χ^2^ tests. For continuous variables, values are mean ± SD or median (interquartile range) given skewness, and *P* values are from *t* test or Wilcoxon rank‐sum (Mann–Whitney) tests as appropriate. Values for maternal BMI, diet, sleep, TV viewing, and physical activity are from data collected when children were about 36 months old. Significant differences are highlighted in bold. South Asian ethnicity includes Pakistani (*n *= 461), Indian (*n *= 43), and Bangladeshi (*n *= 18); white ethnicity includes British (*n *= 370) and other white (*n *= 17). Socioeconomic status groups correspond with Fairley et al. (23) as follows: least deprived (least socioeconomically deprived, most educated and employed, not materially deprived), moderately deprived (employed, no access to money and benefits, not materially deprived), and most deprived (most economically deprived).

^a^ *P* < 0.001.

^b^ *P* < 0.01.

^c^Group differences persisted following adjustment for ethnicity and socioeconomic status.

^d^ *P* < 0.05.

### Associations of TV viewing duration with adiposity using data from all time points

Table 4 contains estimated associations from the mixed effects models, with TV viewing duration modeled as the exposure and adiposity indices as outcomes. Significant interactions between TV viewing duration and age were not evident (Model 1a). There were no significant associations between TV viewing duration and BMI or the sum of skinfolds, regardless of the level of statistical adjustment. However, every 1 h/d of TV viewing was significantly associated with a 0.075‐cm larger (95% CI: 0.0034‐0.15) waist circumference (Model 1; *P* = 0.040). The association was robust to further statistical adjustment for sleep duration, unhealthy snacking, and fruit and vegetable intake (Model 2; *P* = 0.031). All results were unchanged in sensitivity analyses, including adjustment for physical activity level.

**Table 4:** Associations of TV viewing duration with adiposity markers using data from all time points

	**Outcome**	***n* (observations)**	**Overall main effect (Model 1)**	**Interaction effect with age (Model 1a)**	**Main effect at age 18 months** **(Model 1a)**	**Main effect at age 24 months (Model 1a)**	**Main effect at age 30 months (Model 1a)**	**Overall main effect (Model 2)**
**TV viewing (h/d)**	**BMI (kg/m^2^)**	1,325 (3,829)	−0.00042 (−0.030 to 0.029)	0.0024 (−0.00023 to 0.0051)	−0.017 (−0.053 to 0.018)	−0.0030 (−0.033 to 0.027)	0.012 (−0.021 to 0.044)	−0.00070 (−0.031 to 0.029)
	**Sum of skinfolds (mm)**	1,204 (2,578)	−0.058 (−0.18 to 0.067)	−0.0010 (−0.013 to 0.011)	−0.051 (−0.20 to 0.093)	−0.057 (−0.18 to 0.067)	−0.064 (−0.21 to 0.079)	−0.055 (−0.18 to 0.070)
	**Waist circumference (cm)**	1,289 (3,337)	**0.075 (0.0034 to 0.15)^a^**	0.0014 (−0.0050 to 0.0079)	0.065 (−0.019 to 0.15)	**0.074 (0.0018 to 0.15)^a^**	**0.082 (0.0035 to 0.16)^a^**	**0.079 (0.0071 to 0.15)^a^**

Sum of skinfolds constitutes sum of triceps and subscapular thicknesses. Results are regression coefficients (95% CI) and should be interpreted as single‐unit differences in outcomes per 1 h/d of TV viewing. Model 1 adjusted for baseline age and follow‐up time, gender, ethnicity, height (not applicable to BMI), socioeconomic status, maternal pregnancy age, maternal smoking during pregnancy, and maternal BMI. Model 1a is Model 1 with an interaction term between TV viewing duration and follow‐up time. Model 2 is Model 1 but further adjusted for sleep duration, unhealthy snacking, and fruit and vegetable intake. Significant results are highlighted in bold.

^a^ *P *< 0.05.

### Associations of eating meals and snacks when watching TV with adiposity at the 24‐month time point

**Figure 1:**
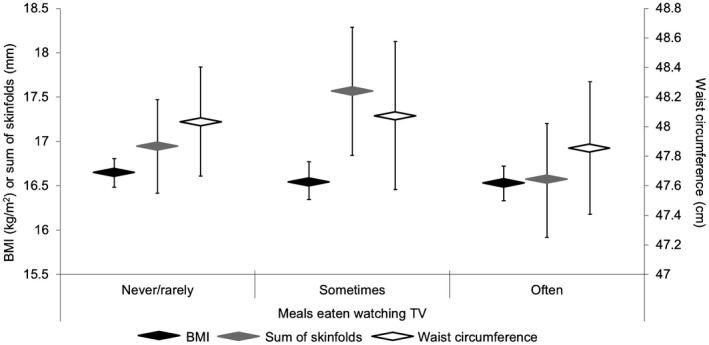
Estimated mean adiposity levels for children aged ~24 months stratified by frequency of eating meals when watching TV. Sum of skinfolds constitutes the sum of triceps and subscapular thicknesses. Sample sizes: never/rarely (BMI: *n* = 354; sum of skinfolds: *n* = 211; waist circumference: *n* = 292), sometimes (BMI: *n* = 199; sum of skinfolds: *n* = 113; waist circumference: *n* = 162), and often (BMI: *n* = 245; sum of skinfolds: *n* = 145; waist circumference: *n* = 203). Results are estimated marginal mean ± 95% CI from linear regression models adjusted for age, gender, ethnicity, height (not applicable to BMI), socioeconomic status, maternal age, maternal smoking in pregnancy, maternal BMI, TV viewing duration, sleep duration, and bedtime regularity. Reference category is never/rarely. Results were unchanged when further adjusted for habitual unhealthy snacking, daily fruit and vegetable intake, and physical activity.

Figures 1and2 show adjusted (Model 2) estimated marginal mean and 95% CI for each adiposity marker by frequencies of eating meals and snacks when watching TV, respectively. Adiposity levels did not vary according to the frequency of eating while watching TV, and the results remained unchanged when habitual unhealthy snacking and daily fruit and vegetable intake were added as covariates. In post hoc analyses, we found no difference in adiposity levels between children who did versus those who did not usually watch TV when eating specific meals (*P* > 0.05 for breakfast, lunch, and dinner) (Supporting Information Figure S4).

**Figure 2:**
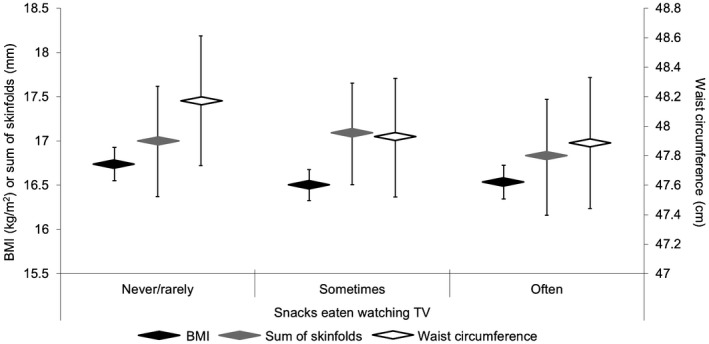
Estimated mean adiposity levels for children aged ~24 months stratified by frequency of snacking when watching TV. Sum of skinfolds constitutes the sum of triceps and subscapular thicknesses. Sample sizes: never/rarely (BMI: *n* = 255; sum of skinfolds: *n* = 152; waist circumference: *n* = 205), sometimes (BMI: *n *= 295; sum of skinfolds: *n *= 179; waist circumference: *n *= 248), and often (BMI: *n *= 248; sum of skinfolds: *n *= 138; waist circumference: *n *= 203). Results are estimated marginal mean ± 95% CI from linear regression models adjusted for age, gender, ethnicity, height (not applicable to BMI), socioeconomic status, maternal age, maternal smoking in pregnancy, maternal BMI, TV viewing duration, sleep duration, and bedtime regularity. Reference category is never/rarely. Results were unchanged when further adjusted for habitual unhealthy snacking, daily fruit and vegetable intake, and physical activity.

### Associations of a TV set in the child’s bedroom with adiposity at the 36‐month time point

**Figure 3:**
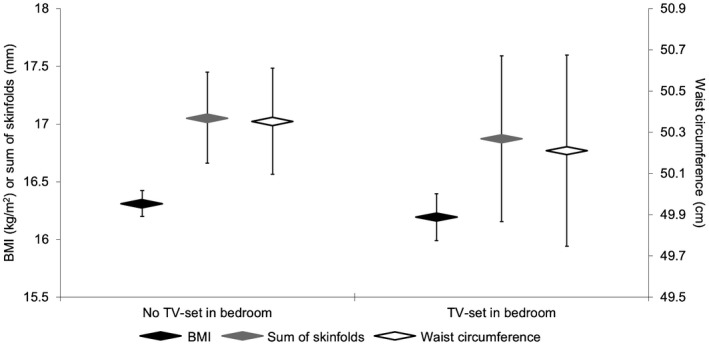
Estimated mean adiposity levels for children aged ~36 months stratified by presence of a TV set in the child’s bedroom. Sum of skinfolds constitutes the sum of triceps and subscapular thicknesses. Sample sizes: no TV in bedroom (BMI: *n *= 688; sum of skinfolds: *n *= 437; waist circumference: *n *= 619) and TV in bedroom (BMI: *n *= 221; sum of skinfolds: *n *= 138; waist circumference: *n *= 201). Results are estimated marginal mean ± 95% CI from linear regression models adjusted for age, gender, ethnicity, height (not applicable to BMI), socioeconomic status, maternal age, maternal smoking in pregnancy, maternal BMI, TV viewing duration, sleep duration, unhealthy snacking, and fruit and vegetable intake. Results were unchanged when further adjusted for physical activity.

Figure 3 shows the adjusted (Model 2) estimated marginal mean and 95% CI for each adiposity marker at ~36 months of age, stratified by the presence or absence of a TV set in a child’s bedroom. There were no differences in adiposity levels regardless of the level of statistical adjustment, and results remained unchanged in sensitivity analyses.

## Discussion

In this study, we incorporated repeated‐measures data spanning approximately 12 to 36 months of age to investigate associations of TV parameters with markers of adiposity in a diverse group of children from a deprived location. Our results provide evidence that TV viewing is associated with larger waist circumference in early childhood, independent of covariates including sleep duration, unhealthy snacking, fruit and vegetable intake, and physical activity level. We found no evidence for associations between eating when watching TV or a TV in a child’s bedroom with adiposity markers.

### Associations of TV viewing duration with adiposity

A recent review of studies in children aged 0 to 4 years acknowledged considerable variability in associations of TV viewing duration with adiposity, particularly across study designs 8. For example, 9 out of 10 longitudinal studies reported at least one significant association between TV viewing duration with adiposity outcomes, whereas results from only 11 out of 22 (generally much smaller) cross‐sectional studies offered comparable results. The authors contend that there may have been insufficient time for the effects of TV viewing to manifest when data from such young children were analyzed cross‐sectionally 8. While plausible, we found little evidence to support this assertion, as there was no strong indication that our associations differed by age (Model 1a). Consistent with other studies 6, 7, 8, we did find that associations varied by adiposity indicators, and although statistically significant, an association between TV viewing and larger waist circumference was small in magnitude. This highlights that large studies with sufficient statistical power may be needed to identify relationships. Every 1 h/d was associated with only a 0.075‐cm larger waist circumference; however, considering this was a study of young children who are on a trajectory of increased daily TV viewing 11, and measurement error may have underestimated the magnitude of our reported associations, it is reasonable to consider that the results may still be clinically meaningful in the long term. Reviews have similarly deliberated that although associations with adiposity tend to be weak, nearly all children watch TV, and therefore a small “effect” across a very large population could be significant for public health 7.

In general, children who watched more TV habitually consumed more unhealthy snacks and fewer fruits and vegetables and exhibited a less favorable sleeping pattern (less overnight sleep). Nonetheless, the association between TV viewing duration and waist circumference remained unchanged even when adjusted for these factors and physical activity (Model 2). Notwithstanding measurement error and possible residual confounding or mediating effects, our data imply that these particular coexisting obesogenic behaviors do not explain the association between TV viewing and young children’s adiposity. While it is possible that other factors that we were unable to investigate (e.g., sleep timing, sleep quality, total caloric intake) may confound or explain our associations, the current results support continued emphasis on curtailing children’s TV time. After just 12 months of age, child TV viewing increases rapidly, and therefore early prevention appears imperative. Key modifiable targets may include reduced maternal TV viewing, limiting the number of hours a TV is on in the home, and heightening parental awareness about the importance of regulating child TV time 11. A TV turnoff week may hold some promise in older children and adolescents 26.

### Associations of eating meals and snacks when watching TV with adiposity

Nearly one‐third of children aged ~24 months often ate meals or snacks while watching TV. Eating when watching TV was related to more TV viewing, greater overall intake of unhealthy snacks, more daytime napping, and less overnight sleep. Similar clustering of adverse energy‐balance‐related behaviors has previously been reported 27. Nonetheless, regardless of the level of statistical adjustment, we found no differences in child adiposity levels according to the frequency of eating while watching TV. This is the first study to explore these associations in early childhood, and as such, a comparison of results with similarly aged children is not possible. In preschool‐aged 28 and primary‐school‐aged children 29, 30, evidence appears to support an association between watching TV during an evening meal and higher BMI, but evidence of equivalent associations for breakfast and lunch is sparse 16. We did consider that some meal‐specific relationships in our population may have been concealed because we incorporated a global measure of meals eaten when TV viewing. Nevertheless, post hoc analysis of food and drinks consumed with specific meals (breakfast, lunch, or dinner) in front of the TV showed no association with adiposity markers (Supporting Information Figure S4). It remains plausible that associations may not be present in infancy but might manifest at later ages either because of a lag or cumulative effect of eating meals when TV viewing. The same could apply to our null results for snacking, but even in older children, it remains unclear whether snacking when watching TV is associated with adiposity 16, 31. An important limitation is that studies have failed to differentiate between consumption of healthy and unhealthy snacks and have not collected information about sugar‐sweetened drinks 32, 33.

### Associations of a TV set in the bedroom with adiposity

Nearly one‐quarter of children had a TV in their bedroom by the age of ~36 months. These children watched more TV in the evening and ate fewer fruits and vegetables compared with children without access to a bedroom TV. Regardless of the level of statistical adjustment, however, there were no observed differences in child adiposity levels according to bedroom TV availability. Having a TV in a child’s bedroom has previously been associated with higher BMI in 3‐ to 4‐year‐old Dutch children 15 and higher risk for overweight in American children aged 1 to 5 years from low‐income families 14. Another study of American children aged 3 to 5 years did not find differences in BMI between children with and without a TV in their bedroom, but that study was small and potentially underpowered 34. In Australian children aged 5 years, girls with overweight, but not boys, were twice as likely than peers with normal weight to have a TV in their bedroom 29. Our null results could be explained by differences between study populations. Our study was uniquely conducted in a younger group of multiethnic children from a deprived city in the United Kingdom. A large European cohort of children aged 11 to 12 years previously indicated heterogeneity of associations between countries 35. It is also noteworthy that the study of American children from low‐income backgrounds 14 took place at the turn of the millennium, prior to a proliferation of mobile devices (laptops, tablets, games consoles, smartphones) that are now widely utilized by children to access media in the home 10. Even so, contemporary studies in older children have continued to report independent associations of having a TV in the bedroom with higher BMI 36 and body fatness 37, 38, including in UK children 39. It is reasonable to hypothesize that an association might manifest in our population when children are older. This could be due to lag or cumulative effects of having access to a TV in the bedroom from a young age or because enforcement of parental rules regarding TV viewing (e.g., no television after a certain time of night) tends to diminish as children get older 40. Prevalence estimates for having a TV in the bedroom increase from infancy to midchildhood 41, and more than half of UK children have a TV in their bedroom by the age of 7 years 39.

### Strengths and limitations

We investigated associations of multiple TV exposures with markers of total and abdominal adiposity in a young and diverse population‐based cohort. As with similar parent‐reported data, TV viewing duration and eating while watching TV are prone to random error and biased reporting, which may have biased our associations toward the null. Parental awareness of children having a TV in their bedroom is arguably less prone to reporting error. However, live and catch‐up TV can now be accessed on various portable devices, meaning that a TV set installation is no longer required to watch TV from the bedroom. Recent data suggested that 41% of 3‐ to 4‐year‐old children in the United Kingdom now watch TV on devices other than a conventional TV screen (mostly a tablet, with 21% of 3‐ to 4‐year‐olds now having their own) 10. By narrowly focusing on TV sets rather than a range of digital platforms that children could use to watch TV in their bedroom, our results may, again, be biased toward the null of no association. Nonetheless, TV sets are still used by UK children more than any other viewing device (primarily for watching live TV rather than increasingly popular video content such as YouTube), and in 2017, there was an increase in the number of hours parents of young UK children said their child watches TV on a TV set 10. Furthermore, our study focused on TV habits, as previous research has shown that TVs, rather than computers, appear to be the type of screen associated with higher child adiposity 37, 39, 42. We included an array of potential covariates and mediators in statistical models, but residual effects likely exist because of measurement imprecision. Finally, direction of association cannot be inferred from these data; it is conceivable that adiposity levels could lead to changes in TV viewing duration rather than vice versa 43. To strengthen claims regarding direction of association and causality, analyses that manipulate subselections of exposure and outcome to investigate early change in the exposure with subsequent change in the outcome are needed.

## Conclusion

In children aged 12 to 36 months, we found that TV viewing duration was associated with larger waist circumference independent of sleep length, dietary factors, and physical activity. Eating while watching TV and a TV in a child’s bedroom were not associated with adiposity at this young age. Limiting children’s TV viewing time may be important for obesity prevention.
