# Supplementary material for: Associations of TV Viewing Duration, Meals and Snacks Eaten When Watching TV, and a TV in the Bedroom with Child Adiposity
Source: Obesity (Silver Spring). 2018 Sep 30;26(10):1619–28. doi: 10.1002/oby.22288 (PMC6207926; doi:10.1002/oby.22288)
Supplement: Supplementary file 1 [file OBY-26-1619-s001.docx]

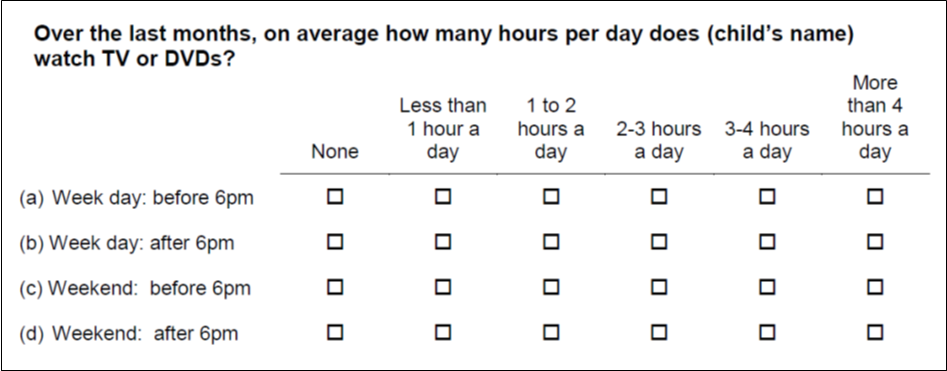


**Figure S1. The interviewer-administered question for parent-reported child TV viewing duration**. Child total daily TV viewing (h/day) was calculated as follows: 1) Average TV viewing before 6pm (h/day) = (((Weekday hours before 6pm × 5) + (Weekend hours before 6pm × 2)) / 7); 2) Average TV viewing after 6pm (h/day) = (((Weekday hours after 6pm × 5) + (Weekend hours after 6pm × 2)) / 7); 3) Total daily TV viewing (h/day) = Average TV viewing before 6pm + Average TV viewing after 6pm. In all formulae, categorical responses were treated as follows: None = 0 h/day; Less than 1 hour a day = 0.5 h/day; 1 to 2 hours a day = 1.5 h/day; 2-3 hours a day = 2.5 h/day; 3-4 hours a day = 3.5 h/day; More than 4 hours a day = 4.5 h/day. Data were collected at the 12, 18, 24 and 36 month time-points.

**
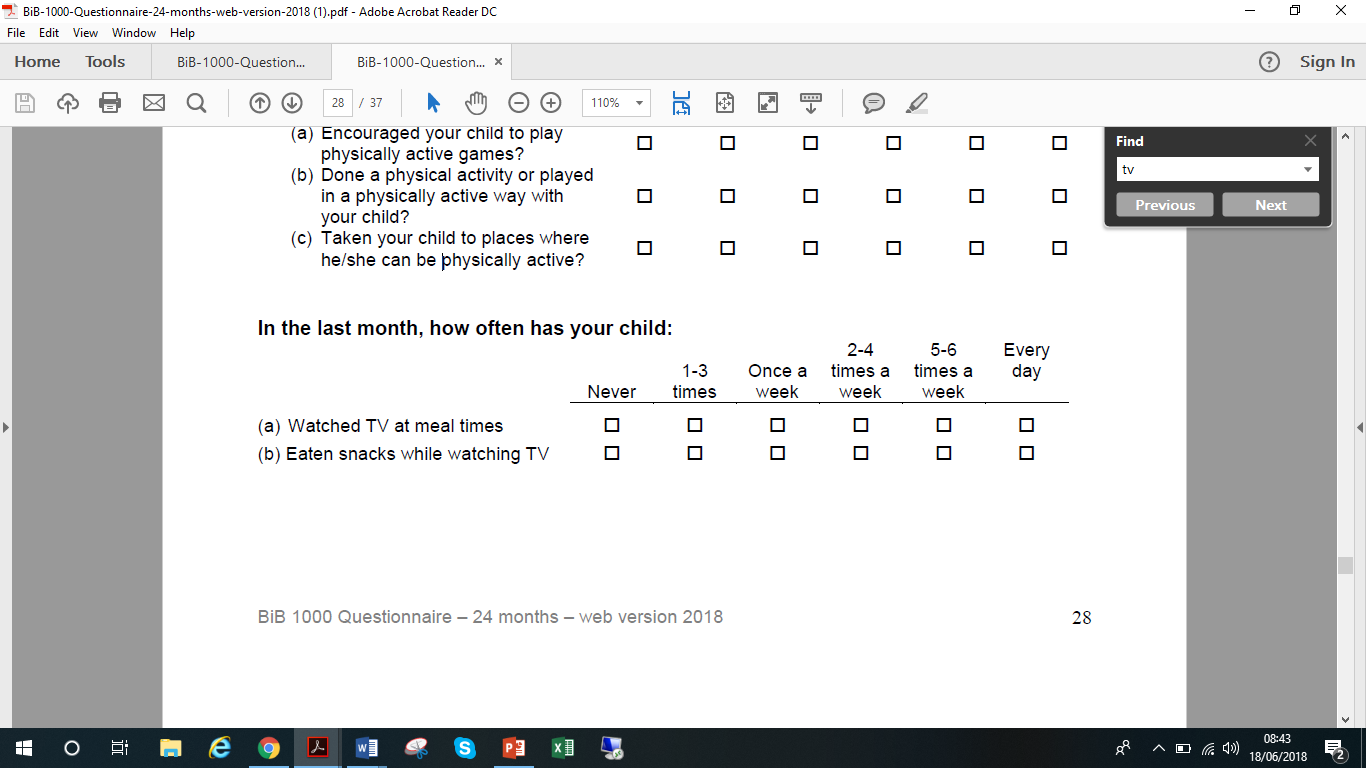
**

**Figure S2. The interviewer-administered question for parent-reported frequency of meals and snacks their child eats when watching TV**. The data were collapsed to three groups respectively for eating meals and snacks whilst watching TV: Never or rarely (≤1-3 times in the last month), Sometimes (1-4 times per week), and Often (≥5 times per week). Data were collected at the 24 month time-point.


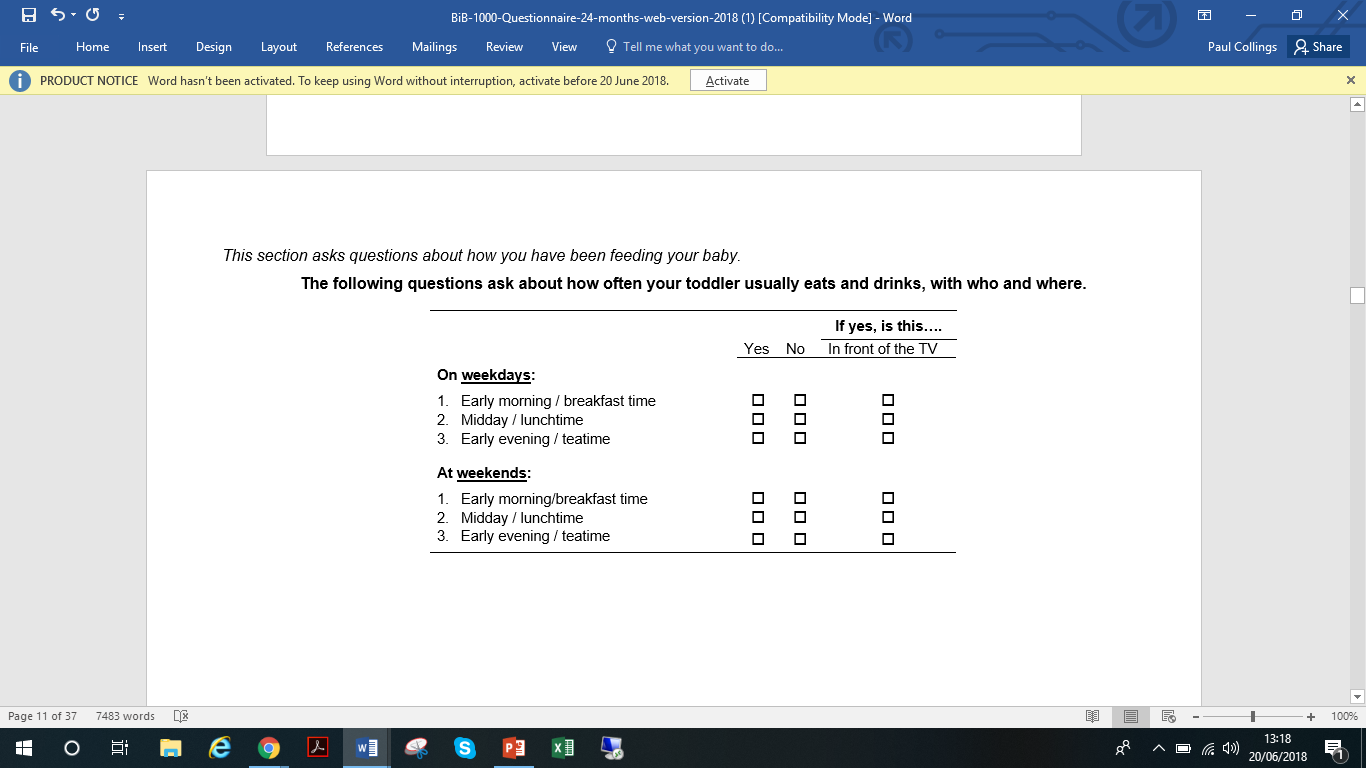


**Figure S3. The interviewer-administered question for parent-reported specific meals (breakfast, lunch, dinner) their child usually eats when watching TV**. Children were classified as usually eating a specific meal whilst watching TV if parents reported that they usually did on weekdays or weekends. Data were collected at the 24 month time-point.


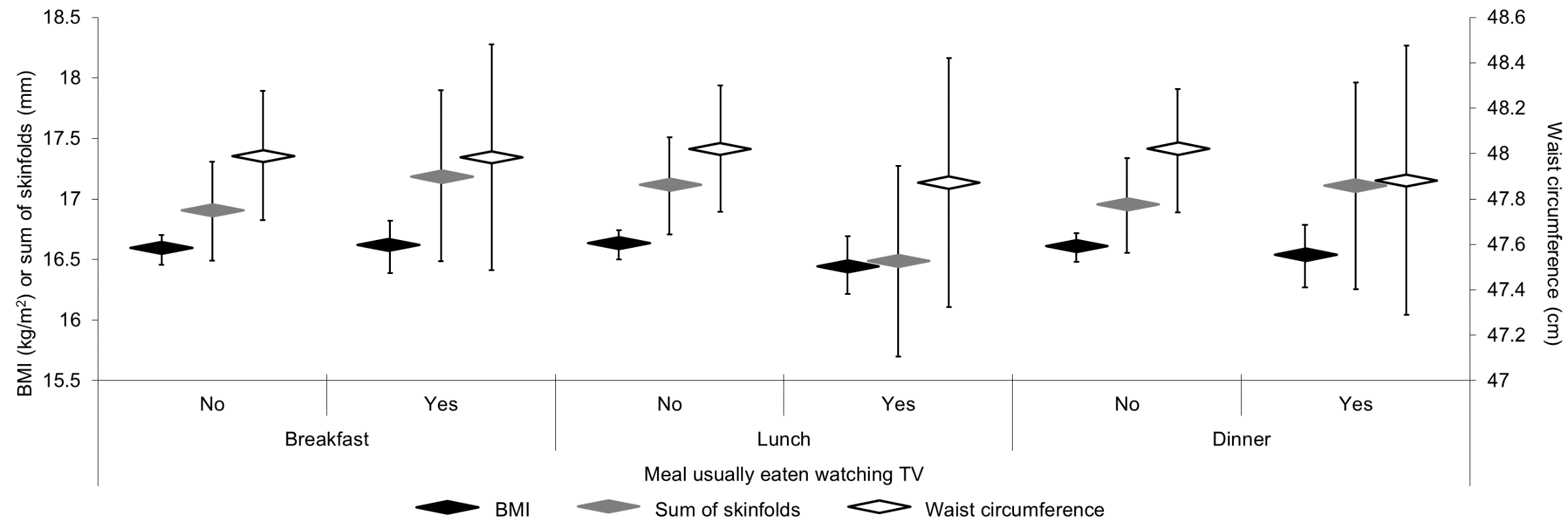


**Figure S4. Estimated mean adiposity levels for children aged ~24 months stratified by specific meals usually eaten watching TV.** Abbreviations: BMI, Body mass index. Sum of skinfolds constitutes the sum of triceps and subscapular thicknesses. Sample sizes: Breakfast (No: BMI: n=596; sum of skinfolds: n=349; waist circumference: n=491; Yes: BMI: n=202; sum of skinfolds: n=120; waist circumference: n=165), Lunch (No: BMI: n=627; sum of skinfolds: n=369; waist circumference: n=516; Yes: BMI: n=171; sum of skinfolds: n=100; waist circumference: n=140), and Dinner (No: BMI: n=653; sum of skinfolds: n=385; waist circumference: n=538; Yes: BMI: n=145; sum of skinfolds: n=84; waist circumference: n=118). Results are estimated marginal means ± 95% confidence intervals from linear regression models adjusted for age, gender, ethnicity, height (not applicable to BMI), socioeconomic status, maternal age, maternal smoking in pregnancy, maternal BMI, and TV viewing duration.
